# Adapting Summer Education Programs for Navajo Students: Resilient Teamwork

**DOI:** 10.3389/fsoc.2021.617994

**Published:** 2021-02-08

**Authors:** Carmella B. Kahn, Heather Dreifuss, Nicolette I. Teufel-Shone, Marissa Tutt, Kelly McCue, Jamie Wilson, Amber-Rose Waters, Kalvina L. Belin, Mark C. Bauer

**Affiliations:** ^1^ Diné College, Shiprock, NM, United States; ^2^ Center for Health Equity and Research, Northern Arizona University, Flagstaff, AZ, United States

**Keywords:** remote learning, online engagement, public health, American Indian, Navajo, summer education program, high school student, undergraduate student

## Abstract

In May 2020, the Navajo Native American Research Center for Health Partnership (Navajo NARCH) was scheduled to launch two summer programs: a 10 weeks-long Summer Research Enhancement Program (SREP) for undergraduate students to learn and practice health research methods and participate in a practicum experience, and a week-long Indigenous Summer Enhancement Program (ISEP) for high school students that introduces a range of health professions and develops leadership qualities. Students accepted into the programs are predominantly Navajo and live within Navajo Nation (NN) during the summer. Due to NN restrictions and CDC guidelines for physically distancing in response to the coronavirus (COVID-19) pandemic, the Navajo NARCH team organized to offer both programs entirely online via Zoom™. This paper explores the instructional teams’ adaptation process to maintain a commitment to preserve the programs’ supportive environment for exploring and developing strong multicultural approaches in public health and health research. In preparation for online instruction, the team developed and offered workshops for staff and instructors to address anticipated challenges. The team identified the following challenges: technological difficulties, social disconnectedness, consistent student engagement, and facilitation of a practicum research experience. Results showed that program adaptations were successful as the team applied collaborative and holistic approaches, and established social connections remotely with students to offer meaningful research and practicum experiences.

## Introduction

American Indians and Alaska Natives (AI/AN) have experienced a disproportionate burden of hospitalizations (five times that of non-Hispanic Whites) and deaths related to coronavirus disease 2019 (COVID-19) (Centers for disease control [CDC], 2020; Kakol et al., 2021). In May 2020, the Navajo Nation (NN) reported the highest per-capita COVID-19 infection rate in the US with 2,304 positive cases per 100,000 citizens compared to the overall United States infection rate of 636.3 positive cases per 100,000 (Silverman et al., 2020). Although the NN population density is low, initial disease transmission was rapid. The NN consists of a 17-million-acre reservation, which extends across three states, New Mexico, Arizona, and Utah; greater than 50%, or approximately 157,000, of the NN’s more than 300,000 enrolled tribal members live within reservation boundaries (Navajo Epidemiology Center, 2013).

To address the infection surge, NN leadership established innovative methods to mitigate the spread of the virus and to communicate COVID-19 response measures and guidelines to citizens within the borders of the NN and beyond. On March 13, 2020, the NN government declared a state of emergency and closed all branches of the government and their services for the remainder of the school year (Becenti, 2020). Since March 29, 2020, the NN Office of the President and Vice President has been using Facebook Live to host weekly virtual town hall sessions, with 9,000–53,000 views per session, to inform citizens on the epidemiology of COVID-19, describe response actions, and provide culturally relevant guidelines to reduce COVID-19 spread within the NN (Nez and Lizer, 2020). On april 8, 2020, the NN President implemented a recurring 57-h weekend curfew, from Friday night through Monday morning, requiring residents to stay in their homes and refrain from large gatherings and trips to stores both on and off the NN (Lambert et al., 2020).

### Setting and Partnership

Against the backdrop of the emerging pandemic, the CDC guidelines for physical distancing, and the NN’s executive orders, the Navajo Native American Research Center for Health Partnership (Navajo NARCH) was preparing for the May 2020 launch of its two annual summer programs: a 10 weeks-long Summer Research Enhancement Program (SREP) for undergraduate students to learn about health research and participate in a practicum experience, and a week-long Indigenous Summer Enhancement Program (ISEP) for high school students that introduces a range of health professions and develops leadership qualities. SREP was established in 2000 and ISEP in 2018. Students accepted into the programs are predominantly Navajo and live on NN during the summer.

The Navajo NARCH is a NIH-NIGMS funded Center led by Diné College (DC) in partnership with Northern Arizona University (NAU). The overall goal of the educational component of the Navajo NARCH is to build the NN’s capacity to improve the health of Navajo and other American Indian (AI) people by increasing the number of professionally trained Navajo practitioners and researchers. Both summer programs use the Diné Educational Philosophy (DEP) as a framework for the curriculum and teaching philosophy for public health education. The DEP is based on the Diné (Navajo) concept of living a long life in wellness and harmony, and being in balance with the natural world and Universe (Benally, 1992; Hughes et al., 2013; Diné College, 2020).

These institutionalized steps for mentoring Navajo students to start a career in health research and public health previously relied on a face-to-face delivery model. The Navajo NARCH instructional teams, consisting of faculty, staff, and teaching assistants, responded to the challenge by offering both programs through a remote, synchronous delivery model via Zoom™, adapting the curricula, and maintaining the reputation, academic rigor, and relational strengths the programs had built over the years. This paper describes the online adaptation process and how the teams maintained the programs’ supportive environment for exploring and developing strong multicultural approaches in public health and health research.

## Methodology

### Participants

#### Indigenous Summer Enhancement Program Recruitment: American Indian High School Students

Recruitment of AI high school students occurred from January 2020 to May 2020. Recruitment involved radio announcements in Navajo and English, social media posts such as Facebook and Instagram, word of mouth, electronic flyers to 20 high schools both on and off the NN, and an email invitation to previous students (to return as peer mentors). Eligibility requirements included current high school enrollment (grades 9–12), parental consent and permission for the student to fully commit to the entire one-week summer program, and a brief online orientation. Fourteen high school students were accepted into the ISEP program in mid-May 2020, including seven peer mentors (previous ISEP students) and seven first time ISEP students. Of the fourteen high school students, one peer mentor and one first time student identified as male, and the other students identified as female. At the time of admission, high school status of those enrolled included: one freshman, three sophomores, seven juniors, and three seniors. All students identified as AI and resided on the NN or in Arizona.

#### Summer Research Enhancement Program Recruitment: American Indian Undergraduate Students

AI undergraduate students were recruited from December 2019 to March 2020. Recruitment involved radio announcements in English and Navajo, applications and flyers mailed and emailed to colleges and universities, and flyers shared through Facebook and college listservs. Eligibility criteria for SREP included being an undergraduate student or a recent graduate from an undergraduate program and commitment to completing the ten-week program. Nine students were accepted into the program; one student identified as male and the other eight identified as female. At the time of admission, undergraduate status of those enrolled include: two sophomores, three juniors, and four seniors. All students identified as AI and resided on the NN or in Arizona.

### Data Collection and Analysis

#### Indigenous Summer Enhancement Program Evaluation

ISEP students completed a 62-item evaluation questionnaire at baseline and at the conclusion of the program. In completing the online survey, students responded to the relevance and understandability of the ISEP content, interest in college preparation, public health knowledge, academic challenges and barriers, further career aspirations, education, and suggested program improvements. Thirteen students completed the baseline survey and 11 students completed the post survey. A NARCH team member who did not mentor or instruct any student assignments administered the evaluation. ISEP evaluation analysis was conducted using Excel^®^ and aggregate data were reported using descriptive statistics.

#### Summer Research Enhancement Program Evaluation

SREP students completed a 73-item evaluation at baseline, at midpoint (week 4), and at the conclusion of the program (week 10). Students responded to questions about the SREP curriculum content and delivery, program or academic challenges and barriers, practicum experience, and interactions with staff, guest speakers, mentors, and instructors. They also reported their knowledge about community health, research, and resilience, and the subjects taught i.e. statistics, program evaluation, research methods, Indigenous research models, and digital storytelling. Nine students completed the baseline and midpoint post survey, and eight students completed the final survey. A total of nine students were admitted into the SREP program; however, due to personal reasons one student did not complete the SREP practicum. A NARCH team member who did not mentor or grade any student assignments administered the evaluation. SREP evaluation analysis was conducted using Excel^®^ and aggregate data were reported using descriptive statistics.

## Results

### Site Description

#### Indigenous Summer Enhancement Program Description

The vision of ISEP at DC is to introduce AI students to careers in public health and health research in order to strengthen research capabilities of tribal colleges and universities. The program is available to high school students grades 9–12, and to returning students as peer mentors. ISEP provides a culturally supportive atmosphere for developing a strong, multicultural approach in public health and health research. The one-week program introduces a range of health professions, teaches digital storytelling, mentorship, and develops leadership qualities with high school students. Peer mentors provide a unique type of mentorship to new ISEP students by leading presentations, activities, and advising students on their projects. Prior to the COVID-19 pandemic, ISEP students convened on the DC Tsaile, AZ campus to experience residential college life, living in dorms, participating in classes, and eating in the cafeteria. In its second year, ISEP ran concurrently with SREP, to encourage direct interactions between high school and undergraduate students.

#### Summer Research Enhancement Program Description

**FIGURE 1:**
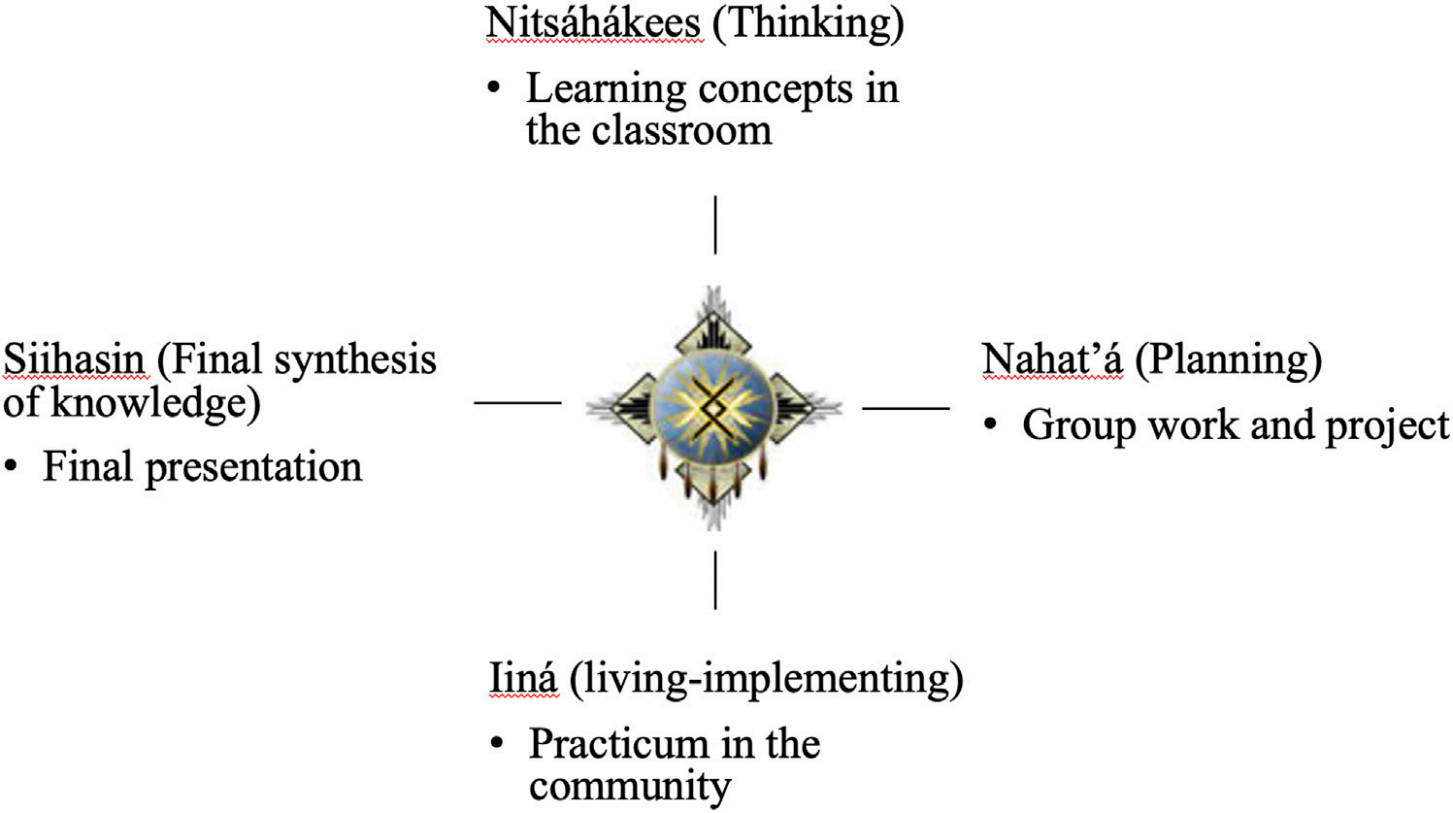
Summer Research Enhancement Program organization based on the Diné Education Philosophy framework.

Prior to the COVID-19 pandemic, SREP for undergraduate students originally included three weeks of course work at the DC Tsaile campus, 6 weeks of site practicum placement in the students’ home communities, and a final week of data analysis and presentations back at Tsaile (Bauer, 2016). Figure 1 illustrates how SREP is organized based on the DEP framework. The course work for the original three weeks focused heavily on research and program evaluation. Digital storytelling training and guest presentations also took place during the three weeks. While at Tsaile, students were encouraged to be physically active and collected personal health data to assess any changes in their BMI or heart rate during the 10-weeks program.

The 6-weeks SREP practicum experience were held in the students’ hometown communities on the NN. Students were typically placed with health programs and research organizations to work 40 h per week for a duration of 6 weeks on community health activities and projects including health fairs, walking/running events, food demonstrations, health education, community needs assessment, etc. Students worked with their site mentor and the SREP practicum coordinator. Students gained hands-on experience with planning community wellness activities, administering community surveys, and assisting with program evaluations. During their practicum, students completed a data-driven research project using data collected or provided by their host site; students would most often analyze program evaluation data. The final week consisted of analyzing the site data and developing PowerPoint presentations as well as finalizing digital stories.

#### Instructional Team and Student Relationships for Indigenous Summer Enhancement Program and Summer Research Enhancement Program

The ISEP and SREP instructional teams and student relationships were strong due to faculty living on campus in the same dorms as students and being present each day in the classrooms. There were opportunities to connect and socialize during mealtimes, in the evenings during exercise times, and on social outings such as hiking or team building at a ropes course. Some instructors for SREP also served as site coordinators and made weekly visits to check on the students and their practicum mentors. During the courses, the instructional teams made themselves available and would often work long hours to ensure students had adequate support. Students were also placed into groups and often formed strong working relationships that fostered group experiences.

### Adaptation Process

#### Indigenous Summer Enhancement Program Adaptations

ISEP online adaptations started with the application itself, which asked students about internet access and availability of a dependable device (i.e., smartphone, tablet, iPad, laptop or desktop computer) for learning purposes. The ISEP staff began transforming the in-person rigorous 11-hour-day schedule to 2, 3-h blocks starting 9:00 am to 12:00 p.m., followed by a 1-h lunch break, and concluding 1:00 pm to 3:00 pm. The staff revised the 2019 schedule to remove the day long team-building activities (e.g., ropes course, Canyon de Chelly hike) and decided by consensus which topics to include in the condensed ISEP schedule. Related topics were combined and converted into 30 min content sessions. New content was introduced in the morning block, while the afternoon block was dedicated for group digital storytelling work. The online version of ISEP was offered June 21 to 26, 2020. See Table 1 for further details on ISEP 2020 adaptations.

**TABLE 1:** Indigenous Summer Enhancement Program adaptions for 2020.

	2019	2020 Adaptations
Location and time	Residential program at DC tsaile campus in Arizona for 1 week	Program took place through zoom™ platform for 1 week
Instructor to instructor interaction	Face-to-face time available	Interaction though zoom™, email, text, slack; flexible
Instructional team/student interactions	Secure rapport with social connectedness	Less secure rapport with fewer times to interact; relative social connectedness
Student to student interaction	In-person opportunity to interact in the classrooms and dorms, and shared exercise hour and meal time	Interaction through slack, text, remind, phone calls, and zoom™ break out rooms
Content delivery	60 min sessions per topic. Course structure is instructor driven	30 min sessions per topic, with five 3-h morning and 3-h afternoon blocks. Uniform structure with warm up, mini- lessons, and student interaction
Content changes	Activities included morning run/walks; community resilience and asset mapping session; social determinants of health game; and indigenous determinants of health session	Begin each day with 15 min check-in, share, and reflection. Zoom™ sessions with indigenous public health leaders and impact of COVID-19 at work sites. Ended each day with 15 min check-out, share, and reflection
Final project	Community and family members invited to in-person presentations of three to 5-min digital stories	Community and family members invited to zoom™ presentations of digital stories
Team building activities	Full day of in person ropes course, canyon de chelly hike, and undergraduate/high school joint activities	Ice breakers, mindfulness, scavenger hunts, stretch breaks

#### Summer Research Enhancement Program Adaptations

SREP was modified to an online format in response to COVID-19. Since the site placements and living in the dorms at DC Tsaile campus would not be possible, the foci became strategies to build and maintain peer and instructional relationships and to shift site practicums to support students to conduct their own research on NN COVID-19 topics. In 2–3 person groups, students developed their own surveys on COVID-19. To support this shift, the coursework for the first four-weeks focused on infectious disease and emphasized data analysis. As in previous years, students were trained in digital storytelling during the five weeks and were instructed to complete their digital stories prior to returning during the last week. The last week was reserved for data analysis and preparing for presentations. The online version of SREP was delivered from May 25th to July 31st, 2020. See Table 2 for further details on SREP 2020 adaptations.

**TABLE 2:** Summer Research Enhancement Program adaptions for 2020.

	2019	2020 Adaptations
Location and time	Three-weeks at DC tsaile campus in Arizona. Six-weeks of site practicum placement in the students’ home communities. Final week at tsaile campus	Online delivery, generally based at home on or off the NN.
Instructor to instructor interaction	Face-to-face content planning. Opportunity to interact in the classrooms and dorms, and exercise hour and meal time	Content planning through zoom™ and email. Primary interaction through text and cell phone
Instructional team/student interaction	Face-to-face classroom instruction. The team worked long and late hours to support student learning. Interaction in classrooms, dorms, exercise hour and meal time	Interaction through zoom™, slack, text, cell phones. The team did not often work late hours but offered office hours for extra support
Student to student interaction	Interaction in the classrooms and dorms, and during exercise hour and meal time	Interaction through zoom™, slack, google Hangout, text, cell phones
Practicum experience	Site placements at home communities with community health focus	Virtual practicum site placement focused on contact tracing
Practicum length	6-weeks	5-weeks
Final research projects	Individual applied research within community health settings. Mentors from practicum sites were assigned to students	Group research on COVID-19. The instructional team served as project mentors for students
Evaluation process	73-Item evaluation on various topics from curriculum to content delivery	Kept the 73-items but added “not Applicable” option to topics that were not administered
Content delivery	3-weeks of coursework. Classroom time started at 9:00 am and ended at 10:00 pm, with 1-h lunch break and 2-h dinner break. Most classes were 1 or more hours	4-weeks of course work. Classroom time started at 9:00 am and ended at 4:00 pm, with 1-h lunch break. Most classes ranged from 30 min to 1 h
Content changes	Content focused on research, intervention development, and program evaluation	Focused on research with emphasis on data analysis, qualitative data collection, and infectious disease. Digital storytelling training took place after the 4 weeks of coursework
Guest presentations	Topics: Food systems, indigenous determinants of health, and traditional medicine and traditional ecological knowledge	Topics: AIAN mental health, COVID-19, and disease etiology and epidemiology of COVID-19 on NN.
Team building activities	Sharing resilience shield, canyon de chelly hike, ropes course, ice breakers, evening exercise hour, just move it 5 k run/walk, and social determinants of health game	Sharing resilience shield, ice breakers, talking circles, and included an adapted online version of the social determinants of health game

In preparation for students to conduct their own research, the schedule provided time for students to meet with their groups to develop research methods, create surveys, and complete protocols to submit to the DC Institutional Review Board (IRB). Table 3 describes the student projects in more detail. The protocols were approved by the IRB in week 6 and students were able to start their data collection. After week 4, students completed training for COVID-19 contact tracing and spent 20 h a week on this activity for 5 weeks.

**TABLE 3:** Summer Research Enhancement Program student COVID-19 projects.

Group project	Purpose	Participants	Methods
Impact of COVID-19 on caregivers	To gather primary data using an online survey to understand the impacts of COVID-19 on caregiver burden, social support, and mental health	*N* = 50	Data collection
			● online survey (survey monkey)
			Data analysis
			● chi-square (OpenEpi, excel^®^)
			Sampling method
			● snowball sampling
			● survey posted on PIs’ facebook accounts
			Inclusion criteria
			● 18 years old or older
			● reside on Navajo nation
			● provide financial supportive care to child (ren), older adults, or person with a chronic illness/disability
			● access to online survey
Impact of COVID-19 Navajo Nation policies	To gather primary data to better understand the impacts COVID-19 has on mental health, behavior, resources, and barriers	Survey	Data collection
		*N* = 111	● online survey (survey monkey)
			● focus group via zoom™
		Focus group	Data analysis
		*N* = 5	● chi-square (VassarStats)
			● *t*-test (excel^®^)
			● grounded theory (focus group)
			Sampling method
			● snowball sampling
			● purposive sampling
			Inclusion criteria
			● 18 years old or older
			● self-identify as AI
			● reside on the Navajo nation
Impact of COVID-19 on Diné College employees	To examine the impacts of COVID-19 on diné college employees, to gain knowledge and perceptions of potential barriers of job duties, and evaluate the support provided to employees	*N* = 66	Data collection
			● online surveys (qualtrics)
			Data analysis
			● chi-square and ANOVA
			Sampling method
			● convenience sampling
			● survey distributed via diné college employee listserv
			Inclusion criteria
			● diné college employee
			● access to internet
			● 18 years and older

Due to the impacts of COVID-19 with health organizations on NN, the SREP practicum was adapted to allow for students to gain public health research experience. Since traditional placement sites were displaced (i.e., reassigned to new areas of their organization to address the public health crisis of COVID-19, working remotely from home or closed until further notice), the SREP director secured an alternate way for students to gain hands-on public health experience as contact tracers for the NN. This was a partnership with DC, the Navajo Department of Health (NDOH), the Navajo Epidemiology Center (NEC), and the Community Outreach and Patient Empowerment (COPE) Program. Students became certified contact tracers by completing the Johns Hopkins University COVID-19 Contact Tracing online course (Coursera, 2020), Health Insurance Portability and Accountability Act (HIPAA) training through Indian Health Services (IHS), and 10 h of live Zoom™ webinars provided by Partners in Health (PIH) prior to beginning their first shifts.

#### Instructional Team Training

In preparation for the online transition, the instructional teams (Table 4) attended a one-day remote training to learn and share strategies for online teaching, Zoom™, and student engagement. Instructors also got feedback on their online teaching style. A plan was put into motion for the teams to serve as mentors for students and were also placed in learning groups with their student mentees.

**TABLE 4:** Indigenous Summer Enhancement Program and Summer Research Enhancement Program instructional team members.

Institution	Teaching experience	Degree(s)	Race	Hometown/work location	Years with the program
DC	Faculty	BA, MA, PhD	White	Farmington, AZ/	21 with SREP
				Shiprock, AZ	
DC	Faculty	BS, MPH, DrPH	AI: Diné	Mariano Lake, NM/	7 with SREP
				Shiprock, AZ	2 with ISEP
DC	Faculty	BA, MS, PhD	White	Manchester, KY/	1 with SREP
				Waterflow, NM	
DC	Teaching assistant	BA	AI: Diné	Shiprock, NM	3 with SREP
					1 with ISEP
University of Colorado	Teaching assistant	BS, MS	AI: Diné	Salina Springs, AZ/	3 with SREP
				Aurora, CO	
NAU	Adjunct faculty	BA, MAT, MPH, DrPH	White	Tucson, AZ	7 with SREP
					3 with ISEP
NAU	Teaching assistant	BA, MPH student	AI: Hopi and Diné	Flagstaff, AZ	1 with SREP
					1 with ISEP
NAU	Adjunct faculty	BS, MPH	AI: Diné	Tuba City, AZ	5 with SREP
					3 with ISEP
NAU	Teaching assistant	BS, MPH	NA: San Carlos Apache	Flagstaff, AZ	2 with SREP
NAU	Teaching assistant	BS, MPH student	AI: Santo Domingo Pueblo (Kewa) and Diné	Kewa, NM	2 with SREP
NAU	Teaching assistant	BS, MPH	AI: Diné	Pinon, AZ/	3 with SREP
				Flagstaff, AZ	2 with ISEP
NAU	Teaching assistant	BS, MPH	White	Flagstaff, AZ	2 with SREP
NAU	Faculty	PhD	White	Williams, AZ/Flagstaff, AZ	15 with SREP

The instructional teams met 1-h each day during the direct instruction stage of the programs. This time was dedicated to reflecting on the day and directly applying real time adjustments in preparation for the next day’s activities. Instructional teams operated on a schedule of flexibility that varied with staff schedules, student absences, changes in curriculum focus, and new opportunities that would arise including availability of guest presenters and contact tracing training.

#### Supportive Environment

Since ISEP and SREP team members overlapped and ISEP began a month after SREP, the ISEP instructors were able to directly apply lessons learned from SREP. Based on these lessons learned, the ISEP team proactively held an orientation for the high school students and their parents to learn about the different platforms used for ISEP. Students learned how to reconnect to Zoom™ via phone if/when there were internet connectivity issues, use break-out rooms, chat and reactions features, share screens and conduct audio checks. ISEP staff demonstrated how to connect to and navigate Blackboard Learn™, DC’s online interface, allowing high school students to experience what it would be like to participate in an online college class. Slack was introduced as a means for peer to peer, small group, and direct communication with the ISEP team outside of Zoom™ classes. The orientation concluded with tips and examples of different ways to successfully set up a student-friendly learning space for the duration of ISEP, complete with hydration and healthy snacks.

The SREP instructional team modified the 10-weeks curriculum to ensure enough course time was offered without overwhelming students with Zoom™ or other online lessons. The instructional teams gave out cell phone numbers for students to schedule meetings or ask questions. The coursework schedule was adjusted so the days started at 9:00 am and ended by 4:00 pm. One SREP instructor provided additional tutoring support for data analysis beyond the scheduled course time. The schedule shifted once the students started contact tracing, with some choosing to do shifts on the weekends or evenings. Students were still expected to complete homework assignments on weekends and after classes ended. The final week was a little challenging because students usually have more contact time but the online platform made it difficult for students and faculty to focus after 4:00 pm. The only exception was when students and faculty stayed online to finish practicing until 1:00 am the day the presentations took place.

#### Multicultural Approaches

Diné traditional knowledge and values were promoted in public health activities such as Hózhó (Kahn-John, 2015), which teaches about the importance of maintaining balance and harmony throughout life by showing respect, having self-disciple and practicing mindfulness in all daily SREP and ISEP activities. Opening and closing prayers were also offered through Zoom™ for ISEP and SREP by a Diné hataalii (Navajo healer) to foster positive thinking and protect students and staff from unbalance and negativity that may arise from research (e.g., death and other sensitive topics). ISEP and SREP guest speakers were invited from various public health and health profession backgrounds. One of the guest speakers for SREP and ISEP presented on the biology of the COVID-19 disease and outcomes. A protection prayer in Navajo and English was given before and after the presentation to ensure the students were not negatively impacted by the information in the presentation.

The DEP was introduced and incorporated into the presentations and activities for ISEP and SREP to teach resilience, public health, research processes, and career pathways to health professions. The incorporation of Diné traditional values helped students understand the curriculum and how to apply their cultural knowledge in public health settings. The SREP talking circles also focused on culturally based topics so students and faculty could discuss their challenges and strengths in addressing academic, professional, or personal issues. The contact tracing created some cultural and emotional issues so the faculty contact tracing coordinator offered time each Monday for students to debrief and also led a talking circle to help address cultural concerns.

### Challenges for Indigenous Summer Enhancement Program and Summer Research Enhancement Program

The team identified the following challenges: technological difficulties, social disconnectedness, inconsistent student engagement, and facilitation of a practicum experience. To address the need for internet access over the large rural expanse of the Navajo Nation, NARCH secured and provided laptops, iPads, and internet hotspots to select faculty and students. More importantly, to create a positive experience firmly grounded in resilience strategies and to counter potential psychological distress, the team incorporated creative techniques to keep students engaged and connected.

#### Technology Issues and Social Disconnectedness

The primary challenges identified by students related to technological difficulties. The majority of SREP and ISEP students lived within the NN, which has limited internet providers and unstable connectivity in many communities. During the programs, a surge of home-based internet users and challenging weather conditions (i.e., monsoons and high winds) resulted in power outages and further affected the internet connections on NN. Students experiencing compromised internet connection were not consistently able to access online resources. Other technological difficulties included software and laptop malfunctions. A student expressed her frustration with technological difficulties, *“[the] software was slow and having bad internet just made it worse.”*

One SREP student stated, *“I had internet problems during the program, but I was able to overcome it and look for other alternative ways to stay connected.”* Students reported overcoming technology barriers by limiting the number of people in their home using the internet during SREP hours, using their phones as internet hotspots, upgrading their internet plans, utilizing the hotspot parking lots or calling into Zoom™ meetings. Calling in with a phone helped students connect but they could not see any lecture material nor fully participate in breakout rooms.

In addition to the aforementioned technological difficulties, students expressed their frustration with being socially disconnected from their peers, mentors, instructors, and community members. Students were asked to share what they liked least about the programs and a SREP student expressed disappointment in being socially disconnected from the community, *“…not being able to work with community programs here in town”*. One ISEP student commented, *“I know that this is out of your hands, but I did not like how we had to do this program virtually.”*

The technology issues may have exacerbated the social isolation, as students were not able to connect with peers and staff on a more personal level. An ISEP student remarked, *“I did not like that it was virtual. I would have loved to physically be in Tsaile at the campus with my peers and teachers. Overall, going virtual was the best option due to our current situation.”* One SREP student shared, *“Part of me wishes we were able to have an in-person SREP because of the strong bonds we were able to create as a peer group. I can't imagine what it must be like in person.”*

#### Student Engagement

Students felt that the SREP and ISEP instructional teams were effective in making the program engaging despite being held virtually. A student expressed her appreciation of the strategies and activities the staff employed to connect with the students, *“The ice breakers were fun, the talking circle was another great component of the program because it allowed us to get to know one another.”* Another student stated, *“[there was] strong team building among the mentors and students.”* Another student felt the activities and social bonding was supportive in the SREP program, *“I love the support from the students and instructors.”*

ISEP students expressed appreciation for meeting other like-minded high school students in an online format during the summer, while they had limited in-person interactions with their peers. One ISEP student commented on the best part of the program, *“I loved the staff and how open everyone was. I like this because the program can be perfectly planned, but it is the people who make it enjoyable. Furthermore, I liked how everyone learned how to work together and the icebreakers.”*

#### Summer Research Enhancement Program Practicum Experience

Despite the changes to the SREP practicum this year, students felt that the practicum experience was valuable. Although the practicum experience was significantly different this year, there was little change in the practicum evaluation responses (Table 5). However, meeting the students’ practicum expectations improved. The entire 2020 SREP cohort strongly agreed that “The SREP practicum met my expectations” in comparison to the 2019 SREP cohort (only 77% respondents strongly agreed to the aforementioned statement).

**TABLE 5:** Evaluation results for 2020 Summer Research Enhancement Program practicum experience.

Evaluation statement	2019 Response (*N* = 13)	2020 Response (*N* = 8)
This practicum provided me basic hands-on experience in research and/evaluation methods in the field	77% strongly agree (10/13)	88% strongly agree (7/8)
	23% agree (3/8)	12% agree (1/8)
During this practicum, I was able to learn about important recent findings regarding community-based public health or chronic disease research, with an emphasis on research pertinent to native American populations of the United States	77% strongly agree (10/13)	62% strongly agree (5/8)
	23% agree (3/13)	38% agree (3/8)
The practicum provided me the opportunity to network with other professionals in the organization and/or in other organizations	85% strongly agree (11/13)	88% strongly agree (7/8)
	15% agree (2/13)	12% agree (1/8)
This practicum provided sufficient opportunity for students to receive feedback on training and research needs	85% strongly agree (11/13)	88% strongly agree (7/8)
	15% agree (2/13)	12% agree (1/8)
The SREP practicum met my expectations	77% strongly agree (10/13)	100% strongly agree (8/8)
	15% agree (2/13)	
	8% neutral (1/13)	

When students were asked to share their perspectives on the practicum, students felt that the practicum was an important component to battling COVID-19, meaningful to patients’ lives, and emotionally challenging for the program staff and patients. One student expressed, *“[I learned to] give empathy to COVID-19 positive patients...to advocate for my patients...I learned critical thinking skills and being able to have more confidence in myself.”* Another student said, *“Contact tracing was an experience I’ll never forget and taught me how to deal with emotional situations.”*

### Resilience in Indigenous Summer Enhancement Program and Summer Research Enhancement Program

#### Indigenous Summer Enhancement Program Resilience Strategies

In the virtual environment, the ISEP program created direct connections between high school students and Indigenous public health leaders, who presented and discussed public health’s role in the COVID-19 pandemic. A member of the Lumbee tribe from North Carolina and faculty member in the Department of Integrative Biology at the University of Colorado Denver presented on the biology of the COVID-19 virus. A Diné leader in the field of genetics presented on conducting ethical and culturally competent genetics research in AI communities. She also discussed her leadership role in her education and research on rural AI veteran suicide prevention. Two other Diné presenters discussed their work in trauma-informed dental care and shared their leadership qualities, including finding purpose in different spaces, speaking up, and being fearless.

Digital storytelling skills served as a collaborative learning tool for students’ final presentations. Students learned about the ethics around digital storytelling, informed consent from people in media, copyright issues in regards to music and images from secondary sources, and citing research sources appropriately. Then staff placed students in small groups, based on interest in a public health discipline, to develop a three to 5 min script that became the narrative soundtrack for the digital stories. Staff provided students with guiding questions to assist in their research of the public health discipline. Students learned and developed digital storytelling technology skills as they recorded the scripts, embedded images, and inserted interviews with Indigenous public health role models to collaborate in real time with their peers to make progress on the final projects. One ISEP student commented, *“I loved meeting new role models and hearing their stories. I would love to meet more in the future.”*

ISEP students shared the following comments on resilience in their evaluations:

“We are resilient in our own ways … ”

“The main thing I learned in ISEP is to always be resilient. I learned this year that I have the capabilities to be a good mentor. This program made me love the public health field even more and I am very grateful to be able to have people and a field that continues to keep me interested and excited to learn.”

“I liked that everyone involved played an amazing role in this program. I got to know a lot of people and make good connections. I believe these connections will last a lifetime and help me better my life and education in the future.”

Table 6 displays the evaluation results for resilience among high school students who participated in ISEP.

**TABLE 6:** Evaluation results for 2019 and 2020 Indigenous Summer Enhancement Program resilience categories.

	2019 (*N* = 14)	2020 (*N* = 11)
Resilience evaluation		
I Can overcome many challenges, I am resilient	100% strongly Agree/Agree	100% strongly Agree/Agree
I Know of leaders in the Navajo nation who have helped improve the health of the Navajo people	100% strongly Agree/Agree	100% strongly Agree/Agree
This program provided me basic hands-on experience in health-related research	100% strongly Agree/Agree	80% strongly Agree/Agree
During the program, I was able to learn about ways I can advocate for the health of my community	100% strongly Agree/Agree	100% strongly Agree/Agree
During the program, I was able to learn about and consider various careers in the health field	100% strongly Agree/Agree	100% strongly Agree/Agree
This week-long experience of the summer enhancement program (SEP) met my expectations	100% strongly Agree/Agree	100% strongly Agree/Agree
Multicultural evaluation		
I feel knowledgeable about my own culture	89% strongly Agree/Agree	90% strongly Agree/Agree
I strongly identify with my cultural heritage	89% strongly Agree/Agree	100% strongly Agree/Agree
I feel knowledgeable about modern medicine and public health	89% strongly Agree/Agree	100% strongly Agree/Agree
Public health and traditional medicine and the incorporation of the two is important to me	100% strongly Agree/Agree	100% strongly Agree/Agree

#### Summer Research Enhancement Program Resilience Strategies

Resilience was demonstrated by the SREP students in their ability to complete the program and effectively learn despite the challenges in completing an online program. Evaluation results from the 2020 cohort compared to the 2019 cohort indicate no difference in knowledge gained, ratings of facilitators and course content, or overall satisfaction in the program. Overall, the majority of students agreed or strongly agreed that the program in 2020 fulfilled these key areas. In addition, a higher proportion of students in 2020 were not intimidated by statistics by the end of the program than students in 2019. In the final evaluation, all eight SREP students agreed that they were resilient and could overcome many challenges.

Both SREP and ISEP students were asked to reflect on the most important thing they learned during the program. Students responded:

“The most important thing I learned so far is that everyone is resilient.”

“Anything is possible with motivation, education, and the best people to give you advice and support … ”

“SREP was able to motivate me to continue pursuing my education. SREP also reignited the passion I have for public health.”

“I have learned to be resilient. Being in a 10-week program [held] virtual[ly] with loads of work to finish is challenging but it is do-able … ”

## Discussion

ISEP and SREP adapted to deliver culturally relevant learning environments for students to explore public health research and future careers, and establish mentor/mentee relationships with staff. The adaptations for ISEP align with changes made in a few other programs offered during the COVID-19 pandemic in terms of shortening the daily time frame and offering more selective content. Of nine regional programs identified for high school students, only four transitioned to an online format during the COVID-19 pandemic. The. University of New Mexico’s Health Careers Opportunity Health Career Academy, typically a 10-weeks, all-day program shortened to 3 h a day for four weeks and provided laptops to students on an as-needed basis (A. Greene, personal communication, May 11, 2020). Dream Keepers, based at New Mexico State University for 10th, 11th and 12th grade students, migrated to a six-week virtual program. The online schedule took place 1 h each weekday, and consisted of two education sessions, personal development, a small group session, and a social hour (Acosta, 2020). The Native Education Forum, a six-day summer program, and Mathematics and Science for Minorities, a three-summer program, also moved to virtual sessions; however, no details were provided for both programs (Andover, 2020; Colorado States University, 2020).

The SREP framework utilizes strategies that are embedded in other pre-COVID-19 research training programs, including internship placements, mentoring, enhancing professional networks, and emphasizing cultural strengths (Salerno et al., 2017; Lee et al., 2018; Taylor et al., 2019). The majority of SREP students indicated in the 2019 post-evaluation they had a positive site practicum experience and gained a tremendous amount of skills and knowledge. In addition, they noted the important role their site mentors played in helping them transition and feel connected. Students also received mentoring from the SREP instructional team. Mentoring not only builds research relationships but also provides for mental and emotional needs, academic support, and career guidance (Salerno et al., 2017; Lee et al., 2018). In addition to promoting mentoring relationships, SREP also builds students’ research networks through sharing final projects with the college community, and offering select students a chance to attend or present at conferences. These research networks help support career development, future research opportunities, and encouragement to continue within the field (Salerno et al., 2017; Lee et al., 2018; Taylor et al., 2019). Students’ cultural strengths are utilized and fostered throughout SREP, particularly by using the DEP framework to guide research and helping students explore topics of resilience and indigenous public health. They often reflected on their identity as indigenous scholars when creating digital stories about individual or community resilience. Helping AI students understand that they can successfully use and create multifaceted identities supports their ability to face new challenges that come from taking on new roles as researchers and scientists (Lee et al., 2018).

The COVID-19 pandemic drastically changed how undergraduate education was delivered in the United States and across the globe (Sandhu and de Wolf, 2020). New and innovative ways of teachings have been applied, with a particular reliance on webinars, Zoom™, and the application of open book exams (Sandhu and de Wolf, 2020). SREP and ISEP students and instructional team members faced numerous challenges to create a summer online program, including grappling with technological difficulties, and supporting the psychological and emotional needs of students that greatly influenced their levels of connection and engagement. The greatest challenge was to adjust the curriculum from 2019 to fit the objectives of the programs while understanding the needs, strengths, and limitations of the students. One major change was the research focus for SREP students to conduct group research projects on COVID-19 through online surveys. A recommended educational strategy to support learning during this crisis is adjusting educational curriculum to fit students’ interest and engage them in applying their knowledge to understanding COVID-19 (Daniel, 2020).

To address technological difficulties, ISEP and SREP instructional teams underwent training to navigate Zoom™, become confident to lead online lectures, and develop a plan to communicate collectively. Institutional programs are encouraged to make necessary adjustments for remote learning, including utilizing asynchronous learning, blogs, and video lessons (Daniel, 2020). Both programs used a modified version to allow a mixture of synchronous and asynchronous learning by using Zoom™ lectures, video recorded lectures, and allowing ample group time in breakout rooms. Zoom™ lectures were also recorded if students indicated they would be missing class (es) for a day or more so they could keep up with the coursework.

The psychological and emotional health of the students were considered when planning the curriculum and delivery methods. The instructional teams understood the mental, emotional, and spiritual strains that the pandemic would have on the programs and participants. A recommendation is for institutional programs to understand that not all home environments are supportive and conducive for study and should focus on providing strong reassurance to students (Daniel, 2020). One study in the US reported undergraduate students were facing increased levels of depression, stress, and anxiety due to COVID-19 (Son et al., 2020). Students reported feeling challenged with social isolation, academic performance, falling behind in research or class projects, and transitioning to online classes (Son et al., 2020). Further, since the NN was hit particularly hard, some SREP students experienced the impacts of COVID-19 on a personal level, grieving the loss of family members from COVID-19; thus, it is critically important to be attentive and understanding to serving students during a crisis. The teams were unable to completely address social disconnection, student engagement, and emotional support, but many efforts were put into play to help alleviate these burdens.

### Lessons Learned

The quick transition to an online platform created unexpected challenges for ISEP and SREP. Some key lessons learned from the summer programs indicate programmatic needs were met through important adaptations and adjustments, and through the cohesive support from the instructional teams. The need for flexibility was evident as the team members reviewed the course curriculum and developed the summer schedule for the programs. Team members stressed the need to manage online engagement to prevent computer strain and stress from prolonged time on computers. At the end of SREP, students and team members noted that more content could have been removed from the curriculum to enable more focused time on key topics and less time on topics that seemed to overload the daily schedule. To address this concern during ISEP, the instructional team employed a strategy to use real time adjustments and do daily check-ins with students to see what worked and did not work so necessary adjustments could be made for the next day’s schedule. Lessons learned for future programmatic needs are to modify the schedules to ensure class time is aligned with key learning objectives and offers flexibility for online or in-person engagement based on students’ feedback during daily check-ins.

The limitations of online engagement increased feelings of social disconnectedness, which the team attempted to offset through innovative methods of interactive activities. Overall, some team members expressed burnout from the online format, which suggests that future programs should include additional team members to divide up content and/or create content teams. Another suggestion is for team members to rotate duties more frequently, which may include getting additional training before the program to take on more roles and responsibilities. One of the challenges with SREP was the program structure where more team members were available the first four weeks, but due to budget constraints only a few stayed on from weeks 5-9, and all team members returned for the final week.

Another adjustment to the longer SREP program was providing more flexibility for students who needed time off or extra support. Some students needed days off to take care of personal and family emergencies more often than would occur in pre-COVID-19 program days. In addition, some of the SREP students were parents and did not always have childcare and faced challenges of being online the full day while watching over their child during the day. Overall, being understanding and patient with life circumstances that occurred due to COVID-19 was key to students’ feeling supported. Lessons learned to support unexpected circumstances for students include creating program structure and guidelines that help students catch up with missed classes and working with students to develop a childcare plan with family members to support their efforts to attend classes during the designated times.

The instructional teams purposefully addressed the emotional, mental, and spiritual issues surrounding COVID-19 while delivering the programs and supporting students. At times, the team admittedly did not feel fully prepared to address the psychosocial needs of the students who needed additional mental health support. Lessons learned for future programs are to involve a team counselor throughout the programs and work with existing college support networks to connect students with mental health resources. Mentors checked in with students daily and the teams utilized cultural-based strategies such as prayer and mindfulness to help alleviate stress. One of the team members also shared resources for bereavement, and guest presenters who specialized in AI mental health were invited to present and share their strategies for promoting mental health during COVID-19. Overall, the primary support for providing flexibility and innovative solutions came from the cohesiveness of the teams and the existing collaborative teamwork dynamics. Lessons learned to support team member dynamics include encouraging positive mindsets, acknowledge students’ needs, and work together to problem solve while maintaining open communication through different platforms.

### Recommendations for Future Program Adaptations

The adaptation process offers implications for future program adaptation to an online environment. First, adaptation needs to be team oriented and approached as collective problem solving that anticipates students’ psychological and physical needs from the onset, as illustrated by the staff and instructor training. Integrating cultural grounded curriculum is supported and recommended in future adaptations from in-person to online summer programs. ISEP and SREP programs are both grounded in the DEP and integrated cultural aspects, such as talking circles and protection prayers that bolstered students’ resilience. An overall supportive environment is recommended to foster a safe learning space that preemptively plans for technology difficulties and social disconnectedness.

## Conclusion

ISEP and SREP succeeded in negotiating the transition to a virtual environment during the COVID-19 pandemic through the use of key resilience strategies that relied on relationships, holistic approaches, and collaboration. First, the online adaptations of the programs emphasized the importance of relationships in Diné culture to build connection between students and between students and the instructional teams, thus overcoming physical distancing by reinforcing social connections. Secondly, the adapted programs used a holistic approach by employing activities that recognized students’ needs to be playful, exercise, pray, and learn. Lastly, the programs applied collaborative team strategies by holding frequent meetings and using consensus decision making. Overall, the online versions of ISEP and SREP can be used as model pipeline support programs to increase the number of AI students who attend college and enter the public health workforce.

## Data Availability

The datasets presented in this article are not readily available because the data belongs to the Navajo Nation, according to the Navajo Research Act and longstanding IRB policy, so any data sharing would have to be specifically approved by them, not by the authors. The datasets are small and include details that could potentially reveal the identity of individual subjects. Requests to access the datasets should be directed to Nicolette Teufel-Shone at Nicky.Teufel@nau.edu.
